# Geographic Disparities by Rural-Urban Status and Drive Time to Care in Tobacco Treatment for COPD

**DOI:** 10.1001/jamanetworkopen.2025.28898

**Published:** 2025-08-26

**Authors:** Arianne K. Baldomero, Anne C. Melzer, Ken M. Kunisaki, Chris H. Wendt, Orly Vardeny, Hildi J. Hagedorn, Steven S. Fu, R. Adams Dudley

**Affiliations:** 1 Pulmonary, Allergy, Critical Care, and Sleep Medicine, Minneapolis VA Health Care System, Minneapolis, Minnesota; 2 Pulmonary, Allergy, Critical Care, and Sleep Medicine, University of Minnesota Medical School, Minneapolis; 3 Center for Care Delivery and Outcomes Research, Minneapolis VA Health Care System, Minneapolis, Minnesota; 4 College of Pharmacy, University of Minnesota, Minneapolis; 5 Department of Psychiatry, University of Minnesota Medical School, Minneapolis; 6 Department of Medicine, University of Minnesota Medical School, Minneapolis

## Abstract

**Importance:**

Many individuals with chronic obstructive pulmonary disease (COPD) who continue to use tobacco do not receive the key intervention of tobacco dependence treatment (TDT). Rurality and drive time to health care services may affect the provision of TDT.

**Objective:**

To examine associations of rurality and drive time to care with provision of TDT among individuals with COPD.

**Design, Setting, and Participants:**

This retrospective cohort study included individuals with COPD who received care from Veterans Affairs (VA) between January 2012 and December 2019. Administrative data from the Veterans Health Administration were used. Individuals with at least 2 encounters with *International Classification Disease *codes for COPD and current tobacco use were included. Data analysis was conducted in October 2023.

**Exposures:**

Rural vs urban home address and drive time to closest VA pulmonary specialty care facility using individual geocoded addresses.

**Main Outcomes and Measures:**

The main outcome was prescription of TDT pharmacotherapy and/or counseling. Multivariable logistic regression models were used to assess associations of rurality and drive time with prescription of TDT.

**Results:**

Among 238 433 individuals with COPD and current tobacco use, the mean (SD) age was 64.1 (9.8) years; 81 189 (93.9%) were male; 2560 (1.1%) were American Indian or Alaska Native, 34 230 (14.3%) were Black or African American, and 185 791 (77.9%) were White. Overall, 97 253 (40.8%) lived in a rural area, and 65 105 (27.4%) had drive times of 61 minutes or longer. Overall, TDT was prescribed to 86 469 individuals (36.3%), but combined pharmacotherapy and counseling only to 10 302 (4.3%). Prescription of any TDT decreased with longer drive times to the closest pulmonary care (eg, 42 324 of 111 126 individuals [38.1%] with drive times ≤30 minutes; 4689 of 14 455 individuals [32.4%] with drive times >120 minutes). In models adjusted for sociodemographic characteristics (age, race and ethnicity, sex, and Area Deprivation Index) and comorbidities, individuals living in a rural area had lower probability of TDT compared with their urban counterparts (34.7% [95% CI, 34.4%-35.0%] vs 37.0% [95% CI, 36.7%-37.2%]). Prescription of TDT steadily decreased from drive time of 30 minutes or less (37.3% [95% CI, 37.0%-37.6%]) to drive times longer than 120 minutes (32.8% [95% CI, 32.1%-33.6%]).

**Conclusions and Relevance:**

In this cohort study of individuals with COPD who smoke, the overall provision of TDT—arguably the most important of all COPD interventions—was low. Additionally, rural residence and longer drive time to specialty care were associated with lower likelihood of receiving TDT. These findings highlight the need to address TDT for all individuals with COPD, especially those facing geographic disparities, to improve health outcomes.

## Key Points

**Question:**

Are rurality and drive time to health care services associated with the provision of tobacco dependence treatment (TDT) among individuals with chronic obstructive pulmonary disease (COPD) who smoke?

**Findings:**

In this cohort study evaluating 238 433 individuals with COPD who smoked tobacco, TDT was provided to 36.3% of participants, but only 4.3% received comprehensive TDT, defined as combined pharmacotherapy and counseling. Individuals living in a rural area had lower probability of TDT compared with their urban counterparts, and the odds of TDT significantly declined with longer drive times to pulmonary specialty care.

**Meaning:**

In this study, TDT among individuals with COPD was low, particularly among those living in rural areas and with longer drive times to pulmonary specialty care.

## Teaser

This cohort study evaluates whether rural residence or physical distance to a specialty clinic are associated with the prescription of comprehensive tobacco dependence treatment among individuals with chronic obstructive pulmonary disease (COPD).

## Introduction

Chronic obstructive pulmonary disease (COPD) is a leading cause of morbidity, mortality, and health care costs across the United States.^1,2,3^ Cigarette smoking is responsible for approximately 80% of COPD cases in the developed world and is the primary modifiable risk factor for both disease onset and progression.^4,5,6,7,8^ Continued smoking after COPD diagnosis leads to worse lung function and outcomes, with individuals actively smoking experiencing an annual decline in forced expiratory flow in the first second of expiration of up to 60 mL/y compared with 30 mL/y in those who quit smoking.^5,9^ Individuals with at least 40 pack-years of smoking have nearly 5 times the risk of severe exacerbations (hazard ratio, 4.98; 95% CI, 3.11-7.97) compared with those who never smoked.^9^ Evidence-based tobacco dependence treatment (TDT) is therefore the most important intervention for individuals with COPD who smoke, and failure to treat tobacco use for individuals with COPD represents a missed opportunity to reduce morbidity, mortality, and health care cost.^10^

Individuals with COPD face significant barriers to cessation, including high nicotine dependence, high prevalence of multimorbidity, mental health and substance use disorders, and low educational attainment.^11,12^ Therefore, they often need extra support from clinicians to quit successfully.^13^ Clinicians play a key role in connecting them to TDT, which is a marker of COPD care quality.^14,15,16^ Comprehensive TDT, combining pharmacotherapy and behavioral counseling, is the gold standard for smoking cessation, yielding higher quit rates than either approach alone (relative risk, 1.83).^15,16,17^ At least 4 counseling sessions of 10 minutes or more are recommended, as increased counseling intensity and frequency improve abstinence rates.^18^

COPD outcomes for rural-residing individuals are worse than for their urban counterparts.^19^ More rural-residing individuals die from or are hospitalized for COPD, with higher burden of poor respiratory status and high symptoms.^20,21,22^ One contributor to these disparities in outcomes may be higher tobacco use rates. Smoking is more prevalent in rural areas, including among adults older than 50 years, who represent the bulk of individuals with COPD.^23^ Higher tobacco use in rural areas may result partly from reduced access to COPD care and TDT. Access to care is a multidimensional issue, and one important barrier is the distance or drive time to health care services. Longer drive times, a key barrier to care, are linked to lower rates of guideline-recommended treatment for both rural and urban patients.^24,25^ In a national Veterans Affairs (VA) cohort, median drive times to care for rural patients were more than twice as long as for urban patients.^24^ However, the impact of drive time on care is not limited to rural patients.^24^ Longer drive times independently reduce receipt of guideline-recommended care for both rural and urban patients.^24^ We hypothesize that geographic barriers to accessing pulmonary specialty care is associated with lower receipt of TDT. To test this, we analyzed a national VA cohort to assess the association of rural residence or drive time to specialty clinics with TDT receipt among veterans with COPD.

## Methods

### Study Oversight

This study was reviewed by and received a waiver of documentation of informed consent from the Minneapolis VA Health Care System institutional review board due to its minimal risk to participants. This cohort study followed the Strengthening the Reporting of Observational Studies in Epidemiology (STROBE) reporting guidelines.

### Data Sources

We used electronic health record data obtained from the national VA Corporate Data Warehouse (CDW), a network of national databases that integrates patient electronic data throughout the VA.^26^ Data for prescription medications for TDT were acquired from the VA Pharmacy Files, while data for tobacco cessation counseling was obtained from VA Outpatient and Inpatient Encounters. We analyzed 2012 to 2019 data to assess TDT before the VA’s 2019 smoke-free campus policy, minimizing confounding from this policy shift.^27^ Data analysis was conducted in October 2023.

### Study Populations

We identified individuals who received care at the VA with a new diagnosis of COPD between January 2012 and December 2019 (eFigure in Supplement 1). Diagnosis and procedure codes used are listed in eTable 1 in Supplement 1. We included individuals who had (1) at least 2 encounters with *International Classification of Diseases, Ninth Revision* (*ICD-9*) or *Tenth Revision* (*ICD-10*), for COPD (*ICD-9*: 490-492.x and 496; *ICD-10*: J40, J41.x, J42, J43.x, and J44)^28^ and were (2) currently using tobacco, identified using tobacco health factors and/or *ICD* codes for tobacco use (*ICD-9*: 305.1; *ICD-10*: F17.x, Z72.0, Z87.891, and O99.33), within 1 year of COPD diagnosis (eTable 1 in Supplement 1).^29^ Tobacco health factor data are generated from clinical reminders during routine health care visits.^29^ This retrievable electronic data field is typically updated at least annually. The tobacco health factor has a specificity of 94.3% for identifying individuals who are currently using tobacco.^30^

### TDT

The primary outcome was provision of any TDT within 1 year of COPD diagnosis. We included all TDT associated with any visit within 1 year from the initial COPD diagnosis. We defined any TDT as prescription for pharmacotherapy and/or counseling for tobacco use cessation (eTable 1 in Supplement 1). We selected a 1-year window after initial COPD diagnosis to capture early TDT, as early intervention slows disease progression, improves lung function, and reduces mortality risk.^10^ TDT pharmacotherapy included (1) nicotine replacement therapy (NRT: gum, lozenge, nasal spray, inhaler, or patch), (2) combination NRT (patch and another form of NRT), (3) bupropion, and (4) varenicline. Counseling for tobacco use was defined using *ICD-9* (V65.42), *ICD-10* (VZ71.6), and *Current Procedural Terminology* codes (eTable 1 in Supplement 1). The secondary outcome was comprehensive TDT, defined as combination pharmacotherapy and counseling.

### Explanatory Variables

Demographic characteristics, geographical location, and comorbid medical and mental health conditions were obtained from the VA CDW. Rural and urban designations for each patient and drive times to the closest VA pulmonary specialty care were obtained from the VA Planning Systems Support Group Geocoded Enrollee Files.^26^

#### Rurality

The VA designates each patient’s home address as urban or rural using Rural-Urban Commuting Area (RUCA) codes, version 2010.^31^ Urban was defined as RUCA codes 1.0 or 1.1 and rural as all others. This definition of urban is narrow, incorporating only census tracts with a metropolitan area core, but we used these VA urban/rural designations for consistency with VA policy and other research.^26,32^

#### Drive Time to the Closest Pulmonary Specialty Care

Drive time estimates from each patient’s home address to the closest VA facility where pulmonary specialty care service was available were calculated using geospatial technologies as described by the VA Health Economics Resource Center. Estimates were based on expected driving routes, traffic, and average driving conditions.^26,33^

#### Covariates

Race and ethnicity data were obtained through patient self-report. We included race and ethnicity as a covariate to adjust for potential confounding, given evidence that racial and ethnic differences may influence disparities in tobacco cessation intervention. We used the Area Deprivation Index (ADI), which provides percentile ranking of neighborhoods by census block groups based on the aggregated domains of income, education, employment, and housing quality, as a surrogate measure for socioeconomic status.^34^ We used the Charlson Comorbidity Index (CCI) to assess health status.^35^ Additionally, we also included specific medical and mental health comorbidities that have been shown to be associated with tobacco use. Cardiovascular comorbidities include coronary artery disease, congestive heart failure, cardiovascular accident, and peripheral vascular disease; and malignant neoplasms include solid organ cancers, hematologic cancers, and melanoma (but not nonmelanoma skin cancers). Substance use disorders include alcohol and illicit drug use (but not nicotine dependence) and serious mental illnesses (SMI) include anxiety, bipolar, mood, posttraumatic stress disorder, personality, and psychotic disorders (eTable 1 in Supplement 1).^36^

### Statistical Analysis

We compared the characteristics of individuals by receipt (yes or no) of TDT. We calculated the percentage of individuals who received the recommended service (a binary outcome at the patient level). Specifically, we calculated the percentage who received any vs no TDT; combined pharmacotherapy and counseling for tobacco use; pharmacotherapy alone; and counseling alone. We also measured the frequency and percentage of TDT by rurality (rural vs urban) and drive time from the patient’s residential address to the closest VA facility where pulmonary specialty care was available (categorized into ≤30 [reference group], 31-60, 61-90, 91-120 and >120 minutes).^24^ We used multivariable logistic regression models to estimate the associations between rurality and drive time to the closest pulmonary specialty care. Average marginal effects were estimated from the logistic regression model by calculating the predicted probability of TDT prescription, averaging the differences across all observations to provide an interpretable measure of association on the probability scale.^37^ Model 1 was unadjusted; model 2 was adjusted for sociodemographic characteristics (age, race and ethnicity, sex, and ADI) and year of COPD diagnosis; and model 3 was adjusted for medical (cardiovascular and malignancy) and mental health comorbidities (substance use disorder and SMI), in addition to covariates included in model 2. Multivariable logistic regression models were adjusted for covariates to minimize the potential confounding effects of sociodemographic factors and clinical factors (full models are shown in eTable 2 and eTable 3 in Supplement 1). Primary analyses evaluated prescription of any TDT, while our secondary analyses evaluated combination pharmacotherapy and counseling (comprehensive TDT). We used an omnibus likelihood ratio χ^2^ test to assess whether rurality and drive time to care were associated with receipt of TDT. We also used likelihood ratio tests to assess whether there is a linear component to the pattern of logs odds of receipt of TDT by drive time to care. Statistical significance was set at *P* < .05, and all tests were 2-tailed. All statistical analyses were performed using SAS software, version 9.4 (SAS Institute).

## Results

Patient characteristics are shown in Table 1. Among 238 433 individuals with COPD and current tobacco use, the mean (SD) age was 64.1 (9.8) years; 81 189 (93.9%) were male; 2560 (1.1%) were American Indian or Alaska Native, 34 230 (14.3%) were Black or African American, 185 791 (77.9%) were White; and 61 854 (25.9%) had a CCI of 3 or higher. More than 30% had cardiovascular comorbidities (80 466 [33.8%]) and diagnosis of serious mental illness (75 461 [31.7%]). Overall, 97 253 (40.8%) lived in a rural area, and 65 105 (27.4%) had drive times to the closest pulmonary specialty care of 61 minutes or longer (Table 1).

**Table 1.** Characteristics of Individuals With COPD Who Smoke by Tobacco Dependence Treatment

Characteristic	Individuals, No. (%)		
	Received tobacco dependency treatment		Total (N = 238 433)
	Yes (n = 86 469)	No (n = 151 964)	
Age, mean (SD), y	61.3 (8.9)	65.8 (10.0)	64.1 (9.8)
Sex			
Female	5280 (6.1)	5537 (3.6)	10 817 (4.5)
Male	81 189 (93.9)	146 427 (96.4)	227 616 (95.5)
Race and ethnicity			
American Indian or Alaska Native	936 (1.1)	1624 (1.1)	2560 (1.1)
Asian	231 (0.3)	519 (0.3)	750 (0.3)
Black or African American	14 253 (16.5)	19 977 (13.2)	34 230 (14.3)
Native Hawaiian or Pacific Islander	586 (0.7)	1042 (0.7)	1628 (0.7)
White	66 047 (76.4)	119 744 (78.8)	185 791 (77.9)
Unknown or declined	4416 (5.1)	9058 (6.0)	13 474 (5.7)
CCI^a^			
0	19 737 (22.4)	33 218 (21.9)	25 594 (22.1)
1-2	47 545 (55.0)	76 443 (50.3)	123 988 (52.0)
≥3	19 551 (22.6)	42 303 (27.8)	61 854 (25.9)
Comorbidities			
Cardiovascular^b^	26 560 (30.7)	53 906 (35.5)	80 466 (33.8)
Malignant neoplasm^c^	7874 (9.1)	17 503 (11.5)	25 377 (10.6)
Substance use disorder^d^	21 146 (24.5)	21 050 (13.9)	42 196 (17.7)
Serious mental illness^e^	34 984 (40.5)	40 477 (26.6)	75 461 (31.7)
Area Deprivation Index, mean (SD), percentile^f^	61.9 ( 24.3)	60.8 (24.3)	61.2 (24.3)
Rurality			
Urban	53 251 (61.6)	87 929 (57.9)	141 180 (59.2)
Rural	33 218 (38.4)	64 035 (42.1)	97 253 (40.8)
Drive time to the closest pulmonary specialty care, min			
≤30	42 324 (49.0)	68 802 (45.3)	111 126 (46.6)
31-60	21 981 (25.4)	40 221 (26.5)	62 202 (26.1)
61-90	11 589 (13.4)	21 546 (14.2)	33 135 (13.9)
91-120	5886 (6.8)	11 629 (7.7)	17 515 (7.4)
>120	4689 (5.4)	9766 (6.4)	14 455 (6.1)

Abbreviation: CCI, Charlson Comorbidity Index.

^a^ CCI scores range from 0 to 33, with higher scores indicating greater disease burden and increased risk of death within 1 year.

^b^ Cardiovascular comorbidities include coronary artery disease, congestive heart failure, cardiovascular accident, and peripheral vascular disease.

^c^ Malignant neoplasm includes solid organ, hematologic, and melanoma; does not include nonmelanoma skin cancers.

^d^ Substance use disorder includes alcohol and illicit drug use; does not include nicotine dependence.

^e^ Serious mental illness includes anxiety, bipolar, mood, posttraumatic stress disorder, personality, and psychotic disorders.

^f^ Area Deprivation Index provides percentile ranking of neighborhoods by census block groups based on the aggregated domains of income, education, employment, and housing quality (percentile ranged from 1 to 100, with higher scores indicating higher levels of socioeconomic disadvantage).

TDT was prescribed to 86 469 participants (36.3% [95% CI, 36.1%-36.5%]), with lower rates among those living in a rural vs urban area (33 218 of 97 253 [34.2%] vs 53 251 of 141 180 [37.7%]) (Figure and Table 2). TDT prescription declined with longer drive times to pulmonary care, from 38.1% among individuals with drive time of 30 minutes or less (42 324 of 111 126) to 32.4% among those with drive time of greater than 120 minutes (4689 of 14 455), irrespective of rural or urban status (Table 2). Comprehensive TDT was prescribed to 10 302 (4.3%) and was similarly lower among individuals living in a rural vs urban area (3474 [3.6%] vs 6828 [4.8%]) and decreased linearly with longer drive times to specialty care (≤30 vs >120 minutes, 5.0% [5597] vs 2.5% [368]) (Table 2). Pharmacotherapy alone was most common (69 318 patients [29.1%]), mainly nicotine patch (23 553 patients [9.9%]), bupropion (18 203 patients [7.6%]), combination NRT (10 056 patients [4.2%]), short-acting NRT (9774 [4.1%]), and varenicline (7732 [3.2%]). Counseling alone was prescribed to 6849 patients (2.9%) (Figure and Table 2).

**Figure.**
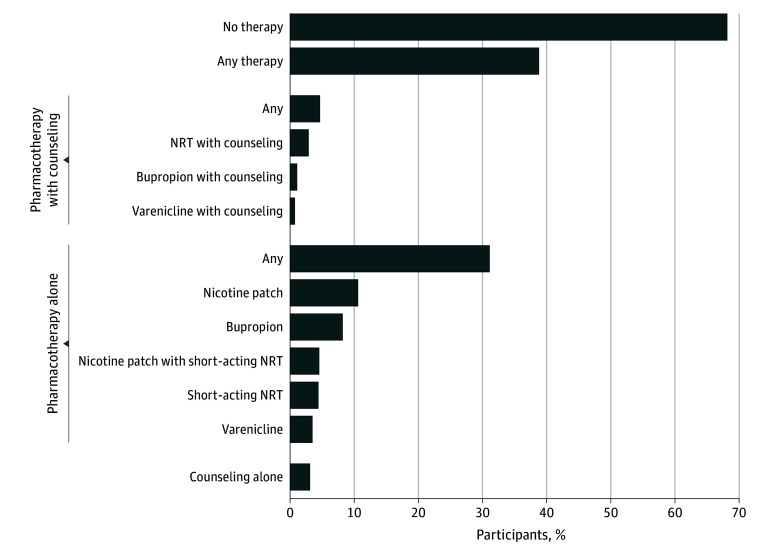
Tobacco Dependence Treatment Among Individuals With Chronic Obstructive Pulmonary Disease Who Smoke NRT indicates nicotine replacement therapy.

**Table 2.** TDT Among Individuals With Chronic Obstructive Pulmonary Disease, Stratified by Rurality and Drive Time to Pulmonary Specialty Care

Treatment	Individuals, No. (%)^a^						
	Residence		Drive time, min				
	Urban (n = 141 180)	Rural (n = 97 253)	≤30 (n = 111 126)	31-60 (n = 62 202)	61-90 (n = 33 135)	91-120 (n = 17 515)	>120 (n = 14 455)
No TDT	87 929 (62.3)	64 035 (65.8)	68 802 (61.9)	40 221 (64.7)	21 546 (65.0)	11 629 (66.4)	9766 (67.6)
Any TDT	53 251 (37.7)	33 218 (34.2)	42 324 (38.1)	21 981 (35.3)	11 589 (35.0)	5886 (33.6)	4689 (32.4)
Pharmacotherapy with counseling	6828 (4.8)	3474 (3.6)	5597 (5.0)	2452 (3.9)	1334 (4.0)	551 (3.1)	368 (2.5)
NRT with counseling	4295 (3.0)	2064 (2.1)	3529 (3.2)	1469 (2.4)	821 (2.5)	327 (1.9)	213 (1.5)
Bupropion with counseling	1509 (1.1)	842 (0.9)	1217 (1.1)	593 (1.0)	309 (0.9)	137 (0.8)	95 (0.7)
Varenicline with counseling	1024 (0.7)	568 (0.6)	851 (0.8)	390 (0.6)	204 (0.6)	87 (0.5)	60 (0.4)
Pharmacotherapy alone	42 052 (29.8)	27 266 (28.0)	33 334 (30.0)	17 740 (28.5)	9288 (28.0)	4927 (28.1)	4029 (27.9)
Nicotine patch	14 228 (10.1)	9325 (9.6)	11 183 (10.1)	6068 (9.8)	3204 (9.7)	1762 (10.1)	1336 (9.2)
Bupropion	10 746 (7.6)	7457 (7.7)	8247 (7.4)	4812 (7.7)	2553 (7.7)	1412 (8.1)	1179 (8.2)
Nicotine patch with short-acting NRT	6527 (4.6)	3529 (3.6)	5379 (4.8)	2447 (3.9)	1219 (3.7)	572 (3.3)	439 (3.0)
Short-acting NRT	6065 (4.3)	3709 (3.8)	4958 (4.5)	2397 (3.9)	1244 (3.8)	641 (3.7)	534 (3.7)
Varenicline	4486 (3.2)	3246 (3.3)	3567 (3.2)	2016 (3.2)	1068 (3.2)	540 (3.1)	541 (3.7)
Counseling alone	4371 (3.1)	2478 (2.5)	3393 (3.1)	1789 (2.9)	967 (2.9)	408 (2.3)	292 (2.0)

Abbreviations: NRT, nicotine replacement therapy; TDT, tobacco dependency treatment.

^a^ Each patient was counted once, prioritizing TDT that was most ideal (ie, combination pharmacotherapy and counseling) in the following order: varenicline with counseling, bupropion with counseling, NRT with counseling, nicotine patch with short-acting NRT, varenicline, bupropion, nicotine patch, short-acting NRT, and counseling alone.

In models adjusting for sociodemographic characteristics and year of COPD diagnosis, rural patients had lower probability of any TDT compared with their urban counterparts (34.4% [95% CI, 34.1%-34.7%] vs 37.2% [95% CI, 36.9%-37.4%]) (Table 3). Models additionally adjusting for comorbidities showed similar lower probabilities of any TDT among individuals living in a rural vs urban area. TDT steadily decreased from drive time of 30 minutes or less (37.3% [95% CI: 37.0%-37.6%]) to drive times of more than 120 minutes (32.8% [95% CI: 32.1%-33.6%]) (*P* < .001 for linear trends).

**Table 3.** Logistic Regression Analyses for Any Tobacco Dependence Treatment Among Individuals With Chronic Obstructive Pulmonary Disease Who Smoke

Characteristic	Patients, No.	Tobacco dependency treatment (N = 238 433)					
		Model 1^a^		Model 2^b^		Model 3^c^	
		Estimated probability, %	Odds ratio	Estimated probability, %	Adjusted odds ratio	Estimated probability, %	Adjusted odds ratio
Rurality^d^							
Urban	141 180	37.4 (37.2-37.7)	1 [Reference]	37.2 (36.9-37.4)	1 [Reference]	37.0 (36.7-37.2)	1 [Reference]
Rural	97 253	34.1 (33.5-34.4)	0.86 (0.85-0.88)	34.4 (34.1-34.7)	0.88 (0.86-0.89)	34.7 (34.4-35.0)	0.90 (0.88-0.92)
Drive time to closest pulmonary specialty care, min^d^^,^^e^							
≤30	111 126	37.8 (37.5-38.1)	1 [Reference]	37.6 (37.3-37.9)	1 [Reference]	37.3 (37.0-37.6)	1 [Reference]
31-60	62 202	35.2 (34.8-35.6)	0.89 (0.88-0.91)	35.5 (35.1-35.9)	0.91 (0.89-0.93)	35.7 (35.4-36.1)	0.93 (0.91-0.95)
61-90	33 135	34.9 (34.4-35.4)	0.88 (0.86-0.90)	34.9 (34.3-35.4)	0.88 (0.86-0.91)	35.2 (34.7-35.7)	0.91 (0.88-0.93)
91-120	17 515	33.4 (32.7-34.1)	0.83 (0.80-0.86)	33.1 (32.4-33.8)	0.81 (0.78-0.84)	33.5 (32.8-34.1)	0.84 (0.81-0.87)
>120	14 455	32.3 (31.5-33.1)	0.79 (0.76-0.81)	32.5 (31.8-33.3)	0.79 (0.76-0.82)	32.8 (32.1-33.6)	0.81 (0.78-0.84)

^a^ Unadjusted.

^b^ Adjusted for age, race and ethnicity, Area Deprivation Index, and year of chronic obstructive pulmonary disease diagnosis. The Area Deprivation Index provides percentile ranking of neighborhoods by census block groups based on the aggregated domains of income, education, employment, and housing quality (percentile ranged from 1 to 100, with higher scores indicating higher levels of socioeconomic disadvantage).

^c^ Adjusted for all covariates in model 2 as well as cardiovascular comorbidities (coronary artery disease, congestive heart failure, cardiovascular accident, and peripheral vascular disease), malignant neoplasms (solid organ, hematologic, and melanoma; does not include nonmelanoma skin cancers), substance use disorder (alcohol and illicit drug use; does not include nicotine dependence), and serious mental illnesses (anxiety, bipolar, mood, posttraumatic stress disorder, personality, and psychotic disorders).

^d^ The omnibus likelihood-ratio χ^2^ test to assess whether rurality and drive time to care were associated with receipt of services was *P* < .001 for all models.

^e^ The likelihood ratio tests to assess whether there is a linear component to the pattern of logs odds of receipt of tobacco dependency treatment by drive time to care was *P* < .001 for all models.

Similarly, comprehensive TDT was lower for individuals living in a rural area (3.7% [95% CI, 3.5%-3.8%]) vs an urban area (4.6% [95% CI, 4.5%-4.7%]) and steadily decreased from drive time 30 of minutes or less (4.8% [95% CI, 4.7%-4.9%]) to drive times of more than 120 minutes (2.6% [95% CI, 2.3%-2.9%]) (*P* < .001 for linear trends) (Table 4). Interaction analysis showed that individuals living in both rural and urban areas experienced decreased TDT as drive time to pulmonary specialty care increased; however, individuals living in urban areas had a higher probability of prescription for TDT at each drive time, with a significantly steeper decline observed in urban areas (*P* < .001) (eTable 4 in Supplement 1).

**Table 4.** Logistic Regression Analyses for Comprehensive (Combination Pharmacotherapy and Counseling) Tobacco Dependency Treatment Among Individuals With Chronic Obstructive Pulmonary Disease Who Smoke

Characteristic	Patients, No.	Tobacco dependency treatment (N = 238 433)					
		Model 1^a^		Model 2^b^		Model 3^c^	
		Estimated probability, %	Odds ratio	Estimated probability, %	Adjusted odds ratio	Estimated probability, %	Adjusted odds ratio
Rurality^d^							
Urban	141 180	4.7 (4.6-4.8)	1 [Reference]	4.7 (4.6-4.8)	1 [Reference]	4.6 (4.5-4.7)	1 [Reference]
Rural	97 253	3.5 (3.4-3.7)	0.74 (0.71-0.77)	3.6 (3.5-3.7)	0.76 (0.73-0.79)	3.7 (3.5-3.8)	0.78 (0.75-0.82)
Drive time to closest pulmonary specialty care, min^d^^,^^e^							
≤30	111 126	4.9 (4.8-5.1)	1 [Reference]	4.9 (4.7-5.0)	1 [Reference]	4.8 (4.7-4.9)	1 [Reference]
31-60	62 202	4.0 (3.8-4.2)	0.78 (0.74-0.82)	4.0 (3.8-4.2)	0.80 (0.76-0.84)	4.1 (3.9-4.3)	0.82 (0.78-0.87)
61-90	33 135	3.9 (3.7-4.0)	0.80 (0.76-0.85)	3.9 (3.8-4.1)	0.82 (0.77-0.87)	4.0 (3.9-4.1)	0.85 (0.79-0.90)
91-120	17 515	3.1 (2.9-3.4)	0.62 (0.57-0.68)	3.1 (2.8-3.3)	0.61 (0.56-0.67)	3.1 (2.9-3.4)	0.64 (0.58-0.70)
>120	14 455	2.5 (2.3-2.8)	0.50 (0.45-0.55)	2.6 (2.3-2.8)	0.51 (0.46-0.57)	2.6 (2.3-2.9)	0.53 (0.47-0.59)

^a^ Unadjusted.

^b^ Adjusted for age, race and ethnicity, Area Deprivation Index, and year of chronic obstructive pulmonary disease diagnosis. The Area Deprivation Index provides percentile ranking of neighborhoods by census block groups based on the aggregated domains of income, education, employment, and housing quality (percentile ranged from 1 to 100, with higher scores indicating higher levels of socioeconomic disadvantage).

^c^ Adjusted for all covariates in model 2 as well as cardiovascular comorbidities (coronary artery disease, congestive heart failure, cardiovascular accident, and peripheral vascular disease), malignant neoplasms (solid organ, hematologic, and melanoma; does not include nonmelanoma skin cancers), substance use disorder (alcohol and illicit drug use; does not include nicotine dependence), and serious mental illnesses (anxiety, bipolar, mood, posttraumatic stress disorder, personality, and psychotic disorders).

^d^ The omnibus likelihood-ratio χ^2^ test to assess whether rurality and drive time to care were associated with receipt of services was *P* < .001 for all models.

^e^ The likelihood ratio tests to assess whether there is a linear component to the pattern of logs odds of receipt of tobacco dependency treatment by drive time to care was *P* < .001 for all models.

## Discussion

In this cohort study of individuals with COPD who smoke, we found evidence of geographic disparities in the provision of TDT associated with rural residence and increasing drive time to specialty care services. Even more worrisome were overall gaps in TDT. Provision of comprehensive TDT, the most effective intervention for tobacco cessation, was very low for all individuals with COPD. Single-agent NRT remained the most common treatment, while varenicline—the most effective pharmacotherapy^17,38^—was the least prescribed. These data highlight the need to target geographic disparities in COPD care to diminish the impacts of COPD on individuals living in rural areas. However, while our findings support that decreased tobacco treatment may contribute to higher tobacco use rates among rural individuals with COPD, they support a large gap in care for COPD in general.

Our results are consistent with our previous work examining other markers of COPD care quality among rural veterans. Individuals in rural areas and those with longer drive times to health care services and specialty care were also less likely to receive spirometry and guideline-adherent inhaler prescriptions.^24,25^ Collectively, these data paint a worrisome picture of disparities in COPD care quality for rural individuals. While the mechanisms through which individuals with COPD in rural areas experience worse outcomes are likely complex, these rural-urban differences in access to health care likely do contribute to worse outcomes.

In general, our observed rates of any TDT were relatively high (36.3%) compared with other studies examining individuals with COPD. Pharmacotherapy prescription rates for patients with COPD who smoke are generally low, ranging from 3% to 16% in outpatient settings, with higher rates (up to 34%) in posthospitalization COPD cohorts.^39,40^ The higher rates we observed in our study likely reflect benefits of the VA system in which TDT medications are widely available and clinicians are prompted to assess interest in cessation at least annually by a clinical reminder. Additionally, the VA delivers TDT through team-based counseling and pharmacotherapy, including all FDA-approved medications and multiple counseling formats (in-person, phone, video). Additional support includes the Quit VET quitline, SmokefreeVET texts, and the Stay Quit Coach app.^41^ Our results are fairly similar to population-based results from Canada, another nationalized health system, where approximately 40% of individuals with COPD reported being provided tobacco cessation medications within the past year.^13^ TDT rates in our study were higher than previous reports but remain low, highlighting a critical gap since tobacco cessation is the most effective intervention for patients with COPD who smoke. Despite some progress, TDT implementation is still far below clinical need, emphasizing the urgent need to prioritize evidence-based strategies.

While we identified disparities in TDT by rurality and drive time to pulmonary specialty, it is worth noting that for all individuals, care provided fell short of what would be optimal care. Optimal TDT for COPD patients who smoke combines pharmacotherapy and behavioral counseling, which more than doubles quit rates.^17^ First-line therapies such as varenicline and combination NRT should be offered.^14,17^

Our study period overlapped the US Food and Drug Administration black box warning that hindered prescribing and acceptance of varenicline, the most effective medication for tobacco treatment.^14^ The proportion of individuals who received counseling and guideline-concordant medications with varenicline or combination NRT was quite low. Although individuals with COPD may have more barriers to receipt of TDT, prior studies confirm that they benefit from tobacco treatment, particularly combined pharmacologic and behavioral treatment, as much as other patients who smoke.^42^

As for many complex care processes, the reasons underlying low participation in TDT are multifactorial and encompass patient, clinician, and system factors. Tobacco cessation and TDT rates vary tremendously from clinician to clinician, even operating within the same health system.^43^ Characteristics of clinicians (eg, training, belief in efficacy of cessation) and clinics (eg, availability of integrated cessation interventions into routine care, accessibility of cessation resources) are strong predictors of receiving cessation support.^44^ Individuals living in rural areas may have fewer nearby clinicians to choose from, and the strain on the health system may be higher in these areas.^45,46^ Primary care clinicians provide most TDT, especially in rural areas with limited pulmonologist access, making them primarily responsible for COPD and TDT management.^47^ Using drive time to pulmonary care as a proxy for access highlights disparities faced by geographically isolated populations. Primary care clinicians often struggle to deliver guideline-based care due to limited resources, time, and specialty support, underscoring the need to strengthen primary care and explore solutions, including telehealth and multidisciplinary teams, to improve TDT and COPD care.^48^ Unfortunately, many clinicians still endorse stigma and fatalism as reasons they do not provide TDT to individuals with COPD.^49^ These individuals are often perceived to be “hardened smokers” who have “given up” on quitting.^34,35^ However, clinicians also support the use of systematic programs, such as proactive tobacco treatment to ensure TDT is equitable and widely available to individuals with COPD.^50^

In addition to clinicians offering treatment and clinics ensuring that treatment is accessible, individuals must accept the treatment. Rural-residing individuals may be at an earlier stage of readiness to quit and could have higher levels of nicotine dependence compared with those living in urban areas.^51,52,53^ If so, interventions to resolve the observed disparities will also need to be tailored to increase patient-level acceptability.

Several studies have reported that programs to improve COPD care quality and outcomes including proactive virtual visits, home monitoring, and remote inhaler teaching and tobacco treatment are effective.^54,55^ Specifically targeting rural individuals with these interventions may be a means to decrease the observed disparities. A key component of launching such initiatives for TDT is ensuring access to high quality counseling in addition to pharmacotherapy, which can be accomplished by increasing use of electronic referrals to quitlines, which are universally available to all veterans and to most of the United States as a whole. Additionally, we recognize that transportation can be a significant barrier to accessing TDT, especially for patients in rural areas or those with limited mobility; addressing transportation barriers and implementing remote interventions are important considerations for improving treatment uptake and outcomes.

### Limitations

Our study has limitations. Reliance on administrative data may have missed some counseling visits or misclassified brief advice as counseling. Spirometry data were unavailable, but we used at least 2 encounter codes, a validated method with 80% to 95% specificity.^20^ We did not have data on whether patients were actually offered or received TDT, so we could not distinguish between whether TDT was provided or whether patients declined it. We lacked data on whether TDT was offered or declined, so we could not distinguish provision from refusal. Results may not generalize beyond veterans, although disparities in an equal-access VA system likely reflect or underestimate those in the broader United States. Care outside the VA was not captured unless medications were VA-provided, but most patients likely received medications through the VA during the study. As an observational study, unmeasured confounding is possible, and causality cannot be established.

## Conclusions

In conclusion, TDT among individuals with COPD was low, particularly for comprehensive TDT. The low TDT was exacerbated by rurality and longer drive time to specialty care. Improving COPD care for all individuals with COPD will require a multidisciplinary approach, with interventions tailored to those with additional geographic barriers to receiving health care services.

## Notes

Supplement 1. **eTable 1. **Diagnosis and Procedure Codes **eTable 2.** Full Models: Logistic Regression Analyses for Receipt of Any Tobacco Dependence Treatment Among Individuals With COPD **eTable 3.** Full Models: Logistic Regression Analyses for Comprehensive (Combination Pharmacotherapy and Counseling) Tobacco Dependence Treatment Among Individuals With COPD **eFigure.** Flow Diagram of Individuals With COPD Who Were Actively Using Tobacco Included in the Analyses **eTable 4.** Interaction Between Rurality and Drive Time to the Closest Pulmonary Specialty Care on Prescription of Any TDT and Comprehensive TDT Among Individuals With COPD

Supplement 2.
Data Sharing Statement
