# Supplementary material for: Geographic Disparities by Rural-Urban Status and Drive Time to Care in Tobacco Treatment for COPD
Source: JAMA Netw Open. 2025 Aug 26;8(8):e2528898. doi: 10.1001/jamanetworkopen.2025.28898 (PMC12381674; doi:10.1001/jamanetworkopen.2025.28898)
Supplement: Supplement 1. — eTable 1. Diagnosis and Procedure Codes eTable 2. Full Models: Logistic Regression Analyses for Receipt of Any Tobacco Dependence Treatment Among Individuals With COPD eTable 3. Full Models: Logistic Regression Analyses for Comprehensive (Combination Pharmacotherapy and Counseling) Tobacco Dependence Treatment Among Individuals With COPD eFigure. Flow Diagram of Individuals With COPD Who Were Actively Using Tobacco Included in the Analyses eTable 4. Interaction Between Rurality and Drive Time to the Closest Pulmonary Specialty Care on Prescription of Any TDT and Comprehensive TDT Among Individuals With COPD [file jamanetwopen-e2528898-s001.pdf]

Supplemental Online Content

Baldomero AK, Melzer AC, Kunisaki KM, et al. Geographic Disparities by Rural-Urban Status and Drive Time to Care in Tobacco Treatment for COPD. *JAMA Netw Open.* 2025;8(8):e2528898. doi:10.1001/jamanetworkopen.2025.28898

eTable 1. Diagnosis and Procedure Codes

eTable 2. Full Models: Logistic Regression Analyses for Receipt of Any Tobacco Dependence Treatment Among Individuals With COPD

eTable 3. Full Models: Logistic Regression Analyses for Comprehensive (Combination Pharmacotherapy and Counseling) Tobacco Dependence Treatment Among Individuals With COPD

eFigure. Flow Diagram of Individuals With COPD Who Were Actively Using Tobacco Included in the Analyses

eTable 4. Interaction Between Rurality and Drive Time to the Closest Pulmonary Specialty Care on Prescription of Any TDT and Comprehensive TDT Among Individuals With COPD

This supplemental material has been provided by the authors to give readers additional information about their work.

© 2025 Baldomero AK et al. *JAMA Network Open*.

eTable 1. Diagnosis and Procedure Codes

	ICD-9	ICD-10	Current Procedural Terminology (CPT)
**Chronic obstructive pulmonary disease (COPD)**	490-492.x, 496	J40, J41.x, J42, J43.x, J44	-
**Tobacco Use**	305.1	F17.208; F17.209; F17.210; F17.213; F17.218; F17.219; F17.290; F17.293; F17.298; F17.299; Z72.0; Z87.891; O99.330; O99.331; O99.332; O99.333; O99.334; O99.335	1032F; 1034F; 1035F; 1036F
**Counseling for Tobacco Use**	V65.42	Z71.6	4000F; 4001F; 4004F; 99407; 0004F; 4000F; C9801; G0375; G0376; 4004F; C9802; G0436; G0437; D1320; G8093; G8402; G9016<br><br>90853; 90832; 90834; 90837; 99212; 99202; 99203; 99204; 99205; 99211; 99212; 99213; 99214; 99215; 99605; 99606; 99607; 99484; 99490-99487; 99489; and a tobacco use ICD/CPT
**Cardiovascular**			
Coronary Artery Disease	410.x, 412.x	I20.0; I20.1; I20.8; I20.9; I21.0-I21.4; I21.9; I21.A; I22.0; I22.1; I22.2; I22.8; I22.9; I23.0-I23.8; I24.0; I24.1; I24.8; I24.9; I25.1-I25.9	-
Congestive Heart Failure	398.91, 402.01, 402.11, 402.91, 404.01, 404.03, 404.11, 404.13, 404.91, 404.93, 425.4–425.9, 428.x	I50.1-I50.4; I50.8; I50.9; I09.81; I11.0; I13.0; I13.2; I125.5; I142.0-I142.9; R57.0	-
Cerebrovascular Accident	362.34, 430.x-438.x	G45.X; G46.X; H34.0; I60.X–I69.X	-
Peripheral Vascular Disease	093.0, 437.3, 440.x, 441.x, 443.1-443.9, 447.1, 557.1, 557.9, V43.4	I70.X; I71.X; I73.1; I73.8; I73.9; I77.1; I79.0; I79.2; K55.1; K55.8; K55.9; Z95.8; Z95.9	-
**Malignancy**<br>Includes solid organ, hematologic, and melanoma; does not include non-melanoma skin cancers	140.x-172.x, 174.x-195.8, 200.x-208.x, 238.6	C00.X-C26.X; C30.X-C34.X; C37.X-C41.X; C43.X; C45.X-C58.X; C60.X-C76.X; C81.X-C85.X; C88.X; C90.X-C97.X	-
**Substance Use Disorder**<br>Includes alcohol and illicit drug use; does not include nicotine dependence	291-292.x, 303-305.x, 648.30–648.34, 965.x.	F10.X-F16.X; F18.X-F19.X	-
**Serious Mental Illness**			

© 2025 Baldomero AK et al. *JAMA Network Open*.

Anxiety Disorder	300.0, 300.2, 300.3, 309.8	F06.4; F40.00-F40.02; F40.10-F40.11; F40.210; F40.218; F40.220; F40.228; F40.230-F40.233; F40.240-F40.243; F40.248; F40.290-F40.291; F40.298; F40.8-F40.9; F41.0-F41.3; F41.8-F41.9; F42.2-F42.4; F42.8-F42.9; F45.20-F45.21; F45.29; F93.0; F60.5	-
Bipolar Disorder	296.0-296.1, 296.4-296.8	F30.X; F31.X	-
Mood/Depressive Disorder	296.2, 296.3, 296.9, 300.4, 311	F32.0-F32.5; F32.9; F33.0-F33.3; F33.40-F33.42; F33.9; F06.31-F06.32; F32.81; F32.89; F32.8; F39; F34.81; F34.89; F34.1; F34.21; F34.9	-
Personality Disorder	301.0-301.9	F60.2-F60.4; F34.0; F60.81	-
Post-Traumatic Stress Disorder	309.81	F43.0; F43.8; F43.10-F43.12	-
Psychotic Disorder	295.0–295.9, 297.1–297.3, 298.8–298.9	F06.0; F06.2; F22; F23; F24; F28; F29; F20.9; F21; F25.0-F25.1; F25.8-F25.9; F60.1	-

Abbreviation: ICD, International Classification of Diseases

© 2025 Baldomero AK et al. *JAMA Network Open*.

eTable 2. Full Models: Logistic Regression Analyses for Receipt of Any Tobacco Dependence Treatment Among Individuals With COPD

Odds Ratio Estimates and Profile-Likelihood Confidence Intervals				
**Effect**	**Unit**	**Estimate**	**95% Confidence Limits**	
**Rurality Rural vs Urban**	1.0000	0.900	0.884	0.917
**Age**	1.0000	0.954	0.953	0.955
**Sex Female vs Male**	1.0000	1.134	1.088	1.182
**Race/Ethnicity Black vs White**	1.0000	1.070	1.043	1.097
**Race/Ethnicity Other/Not Reported vs White**	1.0000	0.907	0.877	0.938
**Area Deprivation Index**	1.0000	1.001	1.000	1.001
**Cardiovascular Yes vs No**	1.0000	0.879	0.857	0.902
**Malignancy Yes vs No**	1.0000	0.892	0.867	0.919
**Substance Use Disorder Yes vs No**	1.0000	1.400	1.368	1.433
**Serious Mental Illness Yes vs No**	1.0000	1.432	1.404	1.460
**Index Diagnosis Year 2012 vs 2019**	1.0000	0.868	0.835	0.902
**Index Diagnosis Year 2013 vs 2019**	1.0000	0.923	0.888	0.958
**Index Diagnosis Year 2014 vs 2019**	1.0000	0.900	0.867	0.934
**Index Diagnosis Year 2015 vs 2019**	1.0000	0.938	0.905	0.973
**Index Diagnosis Year 2016 vs 2019**	1.0000	1.005	0.972	1.039
**Index Diagnosis Year 2017 vs 2019**	1.0000	0.976	0.944	1.009
**Index Diagnosis Year 2018 vs 2019**	1.0000	1.027	0.992	1.062

© 2025 Baldomero AK et al. *JAMA Network Open*.

Odds Ratio Estimates and Profile-Likelihood Confidence Intervals				
**Effect**	**Unit**	**Estimate**	**95% Confidence Limits**	
**Drive Time 31-60 mi vs <=30 min**	1.0000	0.930	0.911	0.950
**Drive Time 61-90 mi vs <=30 min**	1.0000	0.905	0.881	0.930
**Drive Time 91-120 m vs <=30 min**	1.0000	0.835	0.806	0.865
**Drive Time >120 min vs <=30 min**	1.0000	0.810	0.779	0.842
**Age**	1.0000	1.136	1.090	1.184
**Sex Female vs Male**	1.0000	1.071	1.044	1.098
**Race/Ethnicity Black vs White**	1.0000	0.910	0.880	0.941
**Race/Ethnicity Other/Not Reported vs White**	1.0000	1.001	1.000	1.001
**Area Deprivation Index**	1.0000	0.876	0.854	0.898
**Cardiovascular Yes vs No**	1.0000	0.890	0.864	0.916
**Malignancy Yes vs No**	1.0000	1.396	1.364	1.429
**Substance Use Disorder Yes vs No**	1.0000	1.432	1.405	1.460
**Serious Mental Illness Yes vs No**	1.0000	1.136	1.090	1.184
**Index Diagnosis Year 2012 vs 2019**	1.0000	0.881	0.848	0.916
**Index Diagnosis Year 2013 vs 2019**	1.0000	0.934	0.899	0.971
**Index Diagnosis Year 2014 vs 2019**	1.0000	0.909	0.876	0.944
**Index Diagnosis Year 2015 vs 2019**	1.0000	0.945	0.911	0.980
**Index Diagnosis Year 2016 vs 2019**	1.0000	1.011	0.978	1.045
**Index Diagnosis Year 2017 vs 2019**	1.0000	0.980	0.947	1.013
**Index Diagnosis Year 2018 vs 2019**	1.0000	1.029	0.994	1.064

© 2025 Baldomero AK et al. *JAMA Network Open*.

eTable 3. Full Models: Logistic Regression Analyses for Comprehensive (Combination Pharmacotherapy and Counseling) Tobacco Dependence Treatment Among Individuals With COPD

Odds Ratio Estimates and Profile-Likelihood Confidence Intervals				
**Effect**	**Unit**	**Estimate**	**95% Confidence Limits**	
**Rurality Rural vs Urban**	1.0000	0.784	0.750	0.819
**Age**	1.0000	0.965	0.963	0.967
**Sex Female vs Male**	1.0000	1.158	1.066	1.257
**Race/Ethnicity Black vs White**	1.0000	1.176	1.115	1.240
**Race/Ethnicity Other/Not Reported vs White**	1.0000	0.875	0.805	0.949
**Area Deprivation Index**	1.0000	1.002	1.001	1.003
**Cardiovascular Yes vs No**	1.0000	0.985	0.927	1.045
**Malignancy Yes vs No**	1.0000	0.913	0.850	0.979
**Substance Use Disorder Yes vs No**	1.0000	1.474	1.404	1.547
**Serious Mental Illness Yes vs No**	1.0000	1.240	1.186	1.296
**Index Diagnosis Year 2012 vs 2019**	1.0000	0.904	0.828	0.987
**Index Diagnosis Year 2013 vs 2019**	1.0000	0.885	0.810	0.967
**Index Diagnosis Year 2014 vs 2019**	1.0000	0.882	0.809	0.961
**Index Diagnosis Year 2015 vs 2019**	1.0000	0.913	0.839	0.992
**Index Diagnosis Year 2016 vs 2019**	1.0000	0.942	0.872	1.018
**Index Diagnosis Year 2017 vs 2019**	1.0000	1.006	0.932	1.087
**Index Diagnosis Year 2018 vs 2019**	1.0000	1.060	0.981	1.146

© 2025 Baldomero AK et al. *JAMA Network Open*.

Odds Ratio Estimates and Profile-Likelihood Confidence Intervals				
**Effect**	**Unit**	**Estimate**	**95% Confidence Limits**	
**Drive Time 31-60 mi vs <=30 min**	1.0000	0.824	0.784	0.866
**Drive Time 61-90 mi vs <=30 min**	1.0000	0.846	0.794	0.901
**Drive Time 91-120 m vs <=30 min**	1.0000	0.638	0.581	0.699
**Drive Time >120 min vs <=30 min**	1.0000	0.529	0.474	0.590
**Age**	1.0000	0.965	0.963	0.967
**Sex Female vs Male**	1.0000	1.163	1.069	1.262
**Race/Ethnicity Black vs White**	1.0000	1.174	1.114	1.238
**Race/Ethnicity Other/Not Reported vs White**	1.0000	0.880	0.810	0.955
**Area Deprivation Index**	1.0000	1.002	1.001	1.003
**Cardiovascular Yes vs No**	1.0000	0.976	0.919	1.036
**Malignancy Yes vs No**	1.0000	0.906	0.844	0.972
**Substance Use Disorder Yes vs No**	1.0000	1.463	1.393	1.536
**Serious Mental Illness Yes vs No**	1.0000	1.240	1.186	1.296
**Index Diagnosis Year 2012 vs 2019**	1.0000	0.938	0.859	1.024
**Index Diagnosis Year 2013 vs 2019**	1.0000	0.909	0.832	0.993
**Index Diagnosis Year 2014 vs 2019**	1.0000	0.903	0.828	0.984
**Index Diagnosis Year 2015 vs 2019**	1.0000	0.927	0.852	1.008
**Index Diagnosis Year 2016 vs 2019**	1.0000	0.954	0.883	1.032
**Index Diagnosis Year 2017 vs 2019**	1.0000	1.014	0.939	1.096
**Index Diagnosis Year 2018 vs 2019**	1.0000	1.065	0.985	1.150

© 2025 Baldomero AK et al. *JAMA Network Open*.

eFigure. Flow Diagram of Individuals With COPD Who Were Actively Using Tobacco Included in the Analyses

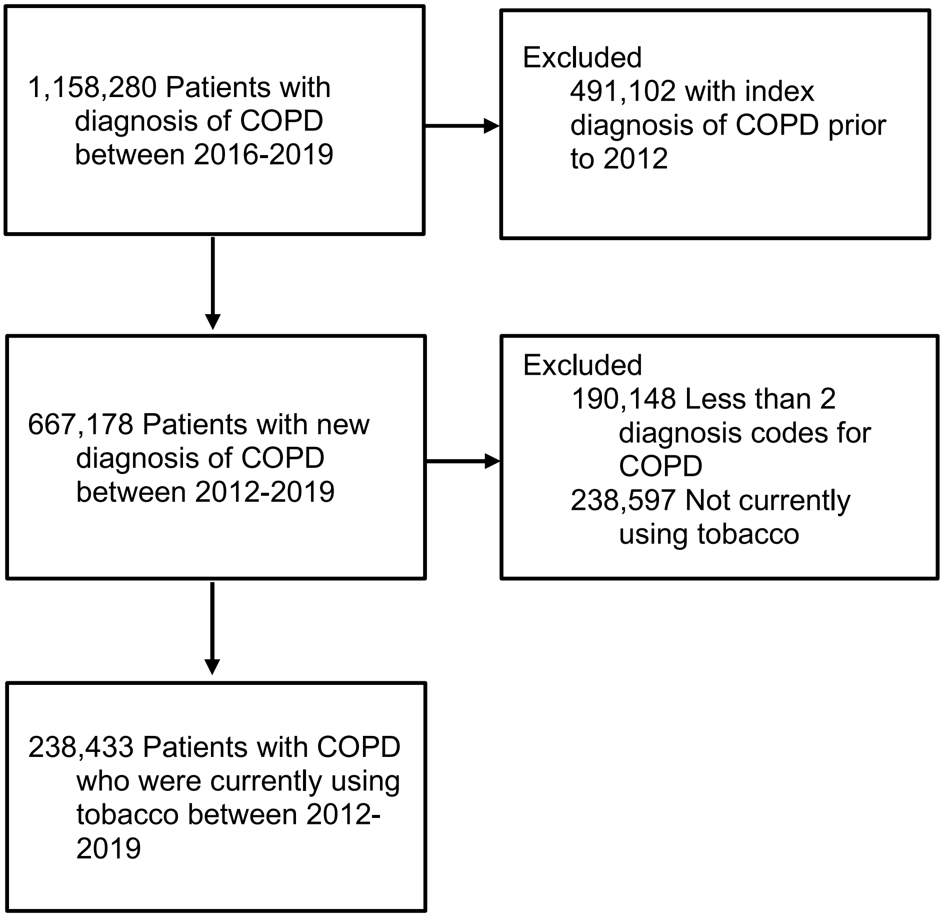

© 2025 Baldomero AK et al. *JAMA Network Open*.

eTable 4. Interaction Between Rurality and Drive Time to the Closest Pulmonary Specialty Care on Prescription of Any TDT and Comprehensive TDT Among Individuals With COPD

	Tobacco Dependency Treatment n=238,433						
	Model 1: Unadjusted			Model 2: Adjusted for Age, Race/Ethnicity, Sex, Area Deprivation Index[a] and Year of COPD Diagnosis		Model 3: Model 2 + Comorbidities (Cardiovascular[b], Malignancy[c], Substance Use Disorder[d], and Serious Mental Illness[e])	
	Patient, No.	Estimated Probability, %	Odds Ratio	Estimated Probability, %	Adjusted Odds Ratio	Estimated Probability, %	Adjusted Odds Ratio
**A. Any TDT [f,g,h]**							
**Rural**							
≤30 min (ref)	18,421	35.3 (34.6-35.9)	1.00	35.3 (34.7-36.0)	1.00	35.4 (34.7-36.0)	1.00
31-60 min	32,377	34.3 (33.8-34.8)	0.96 (0.92-0.99)	34.7 (34.2-35.2)	0.97 (0.94-1.01)	35.2 (34.6-35.7)	0.99 (0.95-1.03)
61-90 min	22,121	34.4 (33.7-34.9)	0.96 (0.92-1.00)	34.5 (33.9-35.1)	0.96 (0.92-1.01)	35.0 (34.4-35.6)	0.98 (0.94-1.03)
91-120 min	13,016	33.1 (32.3-33.9)	0.91 (0.87-0.95)	33.1 (32.3-33.9)	0.90 (0.86-0.95)	33.5 (32.7-34.3)	0.92 (0.87-0.96)
>120 min	10,490	31.7 (30.9-32.6)	0.85 (0.81-0.90)	32.3 (31.4-33.1)	0.87 (0.82-0.91)	32.6 (31.8-33.5)	0.88 (0.83-0.93)
**Urban**							
≤30 min (ref)	95,122	38.3 (38.0-38.6)	1.00	38.1 (37.8-38.4)	1.00	37.7 (37.4-38.0)	1.00
31-60 min	30,281	36.2 (35.6-36.7)	0.91 (0.89-0.94)	36.3 (35.8-36.9)	0.92 (0.90-0.95)	36.3 (35.8-36.9)	0.94 (0.91-0.97)
61-90 min	11,011	35.9 (35.1-36.8)	0.91 (0.87-0.94)	35.4 (34.5-36.2)	0.88 (0.85-0.92)	35.4 (34.5-36.3)	0.90 (0.86-0.94)
91-120 min	4,477	34.4 (33.0-35.8)	0.85 (0.79-0.90)	33.1 (31.7-34.4)	0.79 (0.74-0.85)	33.2 (31.8-34.5)	0.81 (0.76-0.86)
>120 min	3,987	33.7 (32.3-35.2)	0.82 (0.77-0.88)	33.1 (31.6-34.5)	0.79 (0.74-0.85)	33.2 (31.8-34.6)	0.81 (0.75-0.87)
**B. Comprehensive TDT [f,g,h]**							
**Rural**							
≤30 min (ref)	18,421	4.1 (3.8-4.4)	1.00	4.1 (3.8-4.4)	1.00	4.1 (3.8-4.4)	1.00
31-60 min	32,377	3.6 (3.4-3.8)	0.87 (0.79-0.96)	3.6 (3.4-3.8)	0.88 (0.80-0.97)	3.7 (3.5-3.9)	0.90 (0.82-0.99)
61-90 min	22,121	4.0 (3.7-4.3)	0.98 (0.89-1.08)	4.0 (3.8-4.3)	0.98 (0.89-1.09)	4.1 (3.9-4.4)	1.01 (0.91-1.11)
91-120 min	1,3016	2.9 (2.6-3.2)	0.70 (0.62-0.79)	2.8 (2.6-3.1)	0.69 (0.60-0.78)	2.9 (2.6-3.2)	0.70 (0.62-0.80)
>120 min	10,490	2.4 (2.1-2.7)	0.57 (0.49-0.66)	2.4 (2.1-2.7)	0.58 (0.50-0.67)	2.5 (2.2-2.8)	0.59 (0.51-0.69)
**Urban**							
≤30 min (ref)	95,122	5.1 (4.9-5.2)	1.00	5.0 (4.9-5.2)	1.00	4.9 (4.8-5.1)	1.00
31-60 min	30,281	4.2 (4.0-4.4)	0.82 (0.77-0.87)	4.2 (4.0-4.5)	0.83 (0.78-0.89)	4.3 (4.0-4.5)	0.86 (0.80-0.91)
61-90 min	11,011	4.0 (3.6-4.4)	0.78 (0.70-0.86)	3.9 (3.6-4.3)	0.77 (0.70-0.86)	4.0 (3.6-4.3)	0.79 (0.72-0.88)
91-120 min	4,477	3.8 (3.2-4.3)	0.73 (0.63-0.86)	3.6 (3.0-4.1)	0.70 (0.60-0.82)	3.6 (3.1-4.2)	0.72 (0.62-0.85)
>120 min	3,987	2.9 (2.4-3.4)	0.55 (0.46-0.67)	2.9 (2.4-3.4)	0.56 (0.46-0.67)	2.9 (2.4-3.4)	0.57 (0.48-0.69)

[a]Area Deprivation Index provides percentile ranking of neighborhoods by census block groups based on the aggregated domains of income, education, employment, and housing quality (percentile ranged from 1 to 100, with higher scores indicating higher levels of socioeconomic disadvantage).
[b]Cardiovascular comorbidities include coronary artery disease, congestive heart failure, cardiovascular accident, and peripheral vascular disease.

© 2025 Baldomero AK et al. *JAMA Network Open*.

[c]Malignancy includes solid organ, hematologic, and melanoma; does not include non-melanoma skin cancers.
[d]Substance use disorder includes alcohol and illicit drug use; does not include nicotine dependence.
[e]Serious Mental Illnesses include anxiety, bipolar, mood, post-traumatic stress disorder, personality, and psychotic disorders.
[f]The omnibus likelihood-ratio chi-square test to assess whether rurality and drive time to care were associated with receipt of services was $p<0.001$ for all models.
[g]The likelihood ratio tests to assess whether there is a linear component to the pattern of logs odds of receipt of tobacco dependency treatment by drive time to care was $p<0.001$ for all models.
[h]The analysis of the interaction between rurality and drive time to care yielded $p<0.01$ for any TDT and comprehensive TDT across all models, indicating statistically significant interaction effect.

© 2025 Baldomero AK et al. *JAMA Network Open*.
