# Supplementary material for: Geographic Disparities by Rural-Urban Status and Drive Time to Care in Tobacco Treatment for COPD
Source: JAMA Netw Open. 2025 Aug 26;8(8):e2528898. doi: 10.1001/jamanetworkopen.2025.28898 (PMC12381674; doi:10.1001/jamanetworkopen.2025.28898)
Supplement: Supplement 2. — Data Sharing Statement [file jamanetwopen-e2528898-s002.pdf]

Data Sharing Statement

Baldomero. Geographic Disparities by Rural-Urban Status and Drive Time to Care in Tobacco Treatment for COPD. *JAMA Netw Open*. Published August 26, 2025. doi:10.1001/jamanetworkopen.2025.28898

Data
Data available: Yes
Data types: Deidentified participant data
How to access data: Data sets underlying publications will be shared electronically through a de-identified, anonymized dataset. The data will be available through a secure FTP site or sent on encrypted physical media via trackable mail.
When available: beginning date: 11-30-2025

Supporting Documents
Document types: None

Additional Information
Who can access the data: Researchers interested in access to final study data that is not publicly available would need to prepare a brief (i.e., 3-5 page) research proposal, including the specific sample types and data they wish to obtain. Incoming proposals will be screened to ensure that the requests did not overlap or conflict with this project. Data sets will not be shared without first consulting with VA Privacy Officer for approval.
Types of analyses: Researchers interested in access to final study data that is not publicly available would need to prepare a brief (i.e., 3-5 page) research proposal, including the specific sample types and data they wish to obtain. Incoming proposals will be screened to ensure that the requests did not overlap or conflict with this project. Data sets will not be shared without first consulting with VA Privacy Officer for approval.
Mechanisms of data availability: Researchers interested in access to final study data that is not publicly available would need to prepare a brief (i.e., 3-5 page) research proposal, including the specific sample types and data they wish to obtain. Incoming proposals will be screened to ensure that the requests did not overlap or conflict with this project. Data sets will not be shared without first consulting with VA Privacy Officer for approval.
Any additional restrictions: Data sharing will occur under a written agreement that adheres to any applicable informed consent provisions and prohibits the recipient from identifying or re-identifying any individual whose data are included in the dataset. A signed data distribution agreement will be required for each interested researcher, as well as IRB approval, to ensure proper procedures of subject protection.
